# Genetic Variants Associated With Resilience in Human and Animal Studies

**DOI:** 10.3389/fpsyt.2022.840120

**Published:** 2022-05-20

**Authors:** Stephanie Cahill, Tarani Chandola, Reinmar Hager

**Affiliations:** ^1^Evolution, Infection and Genomics, Faculty of Biology, Medicine and Health, Manchester Academic Health Science Centre, The University of Manchester, Manchester, United Kingdom; ^2^Faculty of Humanities, Cathie Marsh Institute for Social Research, The University of Manchester, Manchester, United Kingdom; ^3^Methods Hub, Department of Sociology, Faculty of Social Sciences, The University of Hong Kong, Hong Kong, Hong Kong SAR, China

**Keywords:** resilience, genotype, systematic review, positive adaptation, adversity, gene-environment, animal models

## Abstract

Resilience is broadly defined as the ability to maintain or regain functioning in the face of adversity and is influenced by both environmental and genetic factors. The identification of specific genetic factors and their biological pathways underpinning resilient functioning can help in the identification of common key factors, but heterogeneities in the operationalisation of resilience have hampered advances. We conducted a systematic review of genetic variants associated with resilience to enable the identification of general resilience mechanisms. We adopted broad inclusion criteria for the definition of resilience to capture both human and animal model studies, which use a wide range of resilience definitions and measure very different outcomes. Analyzing 158 studies, we found 71 candidate genes associated with resilience. OPRM1 (Opioid receptor mu 1), NPY (neuropeptide Y), CACNA1C (calcium voltage-gated channel subunit alpha1 C), DCC (deleted in colorectal carcinoma), and FKBP5 (FKBP prolyl isomerase 5) had both animal and human variants associated with resilience, supporting the idea of shared biological pathways. Further, for OPRM1, OXTR (oxytocin receptor), CRHR1 (corticotropin-releasing hormone receptor 1), COMT (catechol-O-methyltransferase), BDNF (brain-derived neurotrophic factor), APOE (apolipoprotein E), and SLC6A4 (solute carrier family 6 member 4), the same allele was associated with resilience across divergent resilience definitions, which suggests these genes may therefore provide a starting point for further research examining commonality in resilience pathways.

## Introduction

Our understanding of how resilience develops is still not well-established largely due to the heterogeneities in the definition of resilience utilized in the past 50 years. Most studies and concept analyses share some commonality in the definition of resilience as the ability to maintain or regain functioning in the face of adversity [e.g., (1–3)]. This broad definition includes both the ability to recover from a stress-related disorder and the ability to withstand adversity, yet these definitions offer little explanation of the mechanisms by which resilience occurs. While there is currently much ongoing work within resilience research to harmonize the definition and quantification of resilience to allow for significant advances in understanding resilience mechanisms (4–7), there is still extensive previous research that can be utilized for significant advances in the identification of underlying resilience mechanisms.

Traditionally, resilience research has been approached from a psychosocial perspective, often focusing on environmental and social factors that influence resilience (8). This has led to reviews with catalogs of so-called “resilience factors” that may be extrinsic or intrinsic to the individual (e.g., high quality care-giving environment, good communication or social skills, self-efficacy or coping) (9–11), with many current studies focussing on genetic resilience factors including twin studies that suggest genetic heritability of resilience accounts for up to 52% of observed variance (12–14). To date, individual genetic and environmental resilience factors, such as specific candidate genes or demographic predictors, have only been identified as weak predictors of resilient outcomes, explaining only a small proportion of the variance and have proven difficult to replicate (7). Furthermore, many of these factors conceptually overlap and mediate, moderate or correlate with one another (15, 16). For example, an individual's genotype is a factor that may affect resilience by influencing an emotional response to stressors. This emotional response to stressors underpins previously identified resilience factors such as emotional regulation, coping or even problem solving. Thus, mapping an individual's genotype may identify a possible resilience mechanism associated with stress response. Such genotype mapping allows for the identification and understanding of the mediating resilience mechanisms (17).

### General Resilience Mechanisms

There are many multifactorial causal pathways that lead to resilient functioning. These pathways involve a complex interplay of biological, social and psychological factors, current experiences and environments, the timing of adversity and experience, and family and community context. However, despite the many factors that may determine if someone is resilient or not, there are potentially fewer distinct biological resilience pathways. Support for this notion comes from psychiatric genetics. For example, a genome-wide association study (GWAS) has indicated that five major psychiatric disorders have a shared risk locus (18), and carrying a “risk” allele of *CACNA1C* confers risk for bipolar disorder, depression, and schizophrenia (19). Calcium Voltage-Gated Channel Subunit Alpha1 C (*CACNA1C)* has been functionally implicated in a broad spectrum of neuropsychiatric disorders, and a recent meta-analysis supports a significant association between *CACNA1C* and major depression disorder (MDD) (20). Similarly, a cross-trait meta-analysis of GWAS identified DCC Netrin 1 Receptor (*DCC)* as a potential risk gene with a pleiotropic role in attention deficit hyperactivity disorder (ADHD), autism spectrum disorder (ASD), depression, and schizophrenia (21).

There is further support of common risk factors leading to diverse psychiatric endophenotypes from studies showing that phenotypes share common features across diverse forms of psychopathology. However, symptoms belonging to specific disorders in early development may not be predictive of a diagnosis of the same disorder later in development but are a marker of more broad adult mental health problems (22, 23). Indeed, there is a paucity of associations between major psychosocial risk factors and specific disorders (24). This lack of association between risks and diagnostic specificity may be explained by the fact that research hypotheses are based on the assumption that disease processes are disorder specific. Psychological disorders are frequently comorbid, with significant overlaps in symptoms. This suggests that resilience research should engage in a transdiagnostic perspective and identify resilience mechanisms that maintain allo/homeostasis (15), rather than focussing on disorder-specific resilience mechanisms. Allo-/homeostatic systems have evolved to maintain functioning despite adversity, therefore, natural selection is more likely to select for mechanisms ensuring allostasis in the face of all stressors, rather than selecting for disorder-specific stress responses. Thus, biological systems that are protective for specific stressors are predicted to be an extension of a more generalized protection system that contributes to maintaining homeostasis within a broad range of environmental disturbances (25, 26).

This dysfunction-specific rationale (15) combined with evidence for frequent comorbidity of stress-related disorders suggests the existence of general resilience mechanisms and, upon breakdown of these mechanisms, stress negatively affects an array of functional systems concordantly. For example, failure to terminate chronic stress-induced activation of the hypothalamus-pituitary-adrenal (HPA) axis can lead to pathological, long-term dysfunction in multiple brain and body compartments (27, 28). Thus, general resilience mechanisms could be those that support flexible HPA axis deactivation. The existence of a resilience mechanism that helps the individual calibrate stress responses optimally, while remaining flexible enough to deploy resources to alternative coping strategies efficiently, would have important consequences for resilience and stress research.

This dysfunction-specific rationale extends to the idea that protection from multiple diseases may be conferred by a limited number of genetic variants of resilience. This rationale is supported by recent research into genetic variants of longevity in non-agenarians and centenarians, which have shown that long-lived individuals (90+ years) have the same number of genetic variants associated with age-related diseases as the general population, yet have an increased prevalence of a small number of novel protective variants (29–32). These few protective variants, in combination with environmental resilience factors, buffer the effect of disease-associated variants and adverse environmental factors, decreasing morbidity at the end of extreme longevity.

A systematic approach that identifies genetic variants conferring resilience is therefore important for several reasons. First, the ability to map genome and phenome relationships more readily onto individual health trajectories by identifying genetic resilience mechanisms brings a health-related perspective to the current more disease-centric genotyping approaches. Further, identifying genetic variants conferring resilience enables effective pharmacotherapy for novel molecular targets. Underlying genetic variants that are associated with general resilience mechanisms may be targeted and thus used to pharmacologically manipulate dysregulated molecules and pathways associated with developing and sustaining symptom severity of trauma-exposed individuals. For example, targeting general resilience mechanisms involved in brain plasticity and cognition could facilitate a change of perspective of trauma exposure and thus lessen the severity of PTSD symptoms (33). The key challenge lies in identifying the common pathways to resilient functioning representing general resilience mechanisms.

### Genetic Variants Conferring Resilience in Humans

Genetic variants, such as allelic variation, can act both as risk and resilience factors. While genetic risk factors increase the likelihood of dysfunction or disease, genetic resilience factors may provide resilience to disease onset or progression (34), or buffer the influence of environmental adversity. However, in the absence of adversity, such genetic resilience factors may appear to have little or no effect. While the role of genetic variants in disease risk has been amply demonstrated (35, 36), much less is known about genetic variants underlying resilience. It is important to note that resilience is not simply the flipside of risk but may co-occur with risk. For example, an individual can experience adversity in a high-risk environment, but also show resilience to a high-risk environment. If exposure and sensitivity to risk factors are high, then the outcome associated with the risk may develop, even in the presence of a resilience factor. Hence the two concepts, risk and resilience, can co-occur. Resilience can also be linked to recovery from stress-related disorders and may not be specific to resisting a specific disease or psychopathology. By focusing on risk factors that predict outcomes requiring diagnostic criteria, studies may not be able to distinguish between individuals who resist developing the disease, and those that recover. Thus, resilience factors may underpin pathways that confer resilience more generally rather than resilience to a particular outcome as defined by current diagnostic criteria.

Most studies on the genetics of resilience in humans have focused thus far on candidate genes with protective variants identified in genes related to serotonergic systems (37), the HPA axis (38), the norepinephrine stress response (39), influencing temporal lobe gray matter volume (40) or amygdala and hippocampal activation to threat (41). While candidate gene approaches have been the mainstay of genomics for over 20 years, increasingly large GWAS have found a lack of robust associations that is seen in candidate gene studies. Among key limitations are lack of statistical power associated with small sample sizes, and potential bias in selecting for candidate genes [e.g., (42–44)]. However, there are some replicable candidate gene x environment (GxE) interactions linked to depression phenotypes within the HPA axis (45) and alcohol use disorder, showing replicability in GWAS with large-effect loci first identified by candidate gene studies (46). Given that resilience is a complex, dynamic process that fundamentally involves both genetic and social/environmental factors, poor replicability across GWAS may be due to the lack of considering both genetic and environmental factors in the study design. While progress on resilience research is advancing, the enormous heterogeneity in the definition of resilience also precludes reproducibility and undermines the robustness of results across studies, ultimately hindering significant advances in the development of effective interventions based on general resilience mechanisms. By focusing on specific genetic variants, we are better able to identify common biological resilience pathways and potential targets for intervention.

### Animal Models in the Study of Resilience

The candidate gene approach used in studies on the genetics of human resilience is replicated in genetic animal models of resilience using a targeted approach. Due to ethical reasons, experimental investigations of the genetic effects of resilience, especially regarding the central nervous system, can only be conducted in animals. Many recent animal models have been specifically designed to distinguish between vulnerable and resilient phenotypes, through a variety of approaches.

One approach is to use transgenic models where animal genomes (predominantly rodent) are modified, resulting in knock out (KO) or knock in of specific candidate genes. Several studies using such models have now demonstrated the influence of single genetic variants on stress vulnerability or resilience (47). However, the limitations of using transgenic animal models are important to consider. Transgenic models can provide information about the general function of the specific gene targeted, yet resilience, like many spectrum disorders, is multigenic. Thus, linkage analyses may be a more appropriate approach to detect variants associated with resilience phenotypes [e.g., (48)]. It is also worth noting that genes often play different roles or may potentially even have opposite functions in separate tissues or cell types [e.g., (49)]. Further, the developmental time frame during which gene expression is modulated also needs to be considered, due to the vital influence of developmental timings on the maturation of different parts of the stress system on later stress vulnerability or resilience (50).

Selective breeding of a specific phenotype is another approach used in animal models of resilience. An example of this is the stress reactivity mouse line (51). Starting from a population of outbred mice, two breeding lines were established according to the outcomes of a stress reactivity test. Mice exhibiting hypo- or hyperactivity of the HPA axis, measured *via* corticosterone levels in blood, were selected for high or low reactivity breeding lines. By the first generation, the breeding line has diverged, allowing for further characterization and investigation of the underlying molecular principles. In this model, the high reactivity breeding line shows cognitive deficits, restlessness, and agitation, which is often seen in melancholic depression (51), and also exhibit analogous neuroendocrine activity and morphometric differences (52). Using such models in genome-wide association studies can lead to the identification of novel genes or networks. Selective breeding also has the potential to identify causal genetic factors using careful research designs, as the phenotypic differences are present before exposure to any stressor.

Similar to the selective breeding approach, the intrinsic heterogeneity of whole populations can be used by grouping animals into subpopulations dependent upon their performance in a behavioral assessment, such as the forced swim test. For example, in a rat model, Wong et al. (53) used chronic restraint stress (CRS) to elicit a depressive-like behavior in the forced swim test, measured by increased floating time/immobility. However, not all animals develop this phenotype, so the cohort is separated into CRS resilient and CRS non-resilient animals, allowing for the identification of genetic differences in these subphenotypes. While this approach has the same advantages as the selective breeding approach, measuring an analogous phenotype with high translatability to human behavior is challenging [e.g., (54)], with some authors arguing that anthropomorphic interpretations of rodents behavior does not accurately model depression or resilience to depression (55).

The main question regarding all animal models of resilience is how they relate to resilience as defined in humans. Since there is no standard definition of resilience in humans, animal models of resilience therefore use different paradigms and outcome variables to measure resilience, analogous to the different definitions and operationalisation of resilience in human studies. Despite the limitations of animal models of resilience, as with the human studies, our broad inclusion criteria enable us to capture the diversity of paradigms used in animal models and aim to identify commonalities in genetic variants underlying resilience.

### Differences in Environmental Sensitivity

One of the fundamental concepts of resilience research is that individuals differ in their sensitivity to environmental influences, with increasing evidence that certain genetic variants confer increased environmental sensitivity (56–59). These individual differences in behavioral responses to the environment are seen across species, from zebra finches to mice, non-human primates to humans (60). This concept is traditionally framed within psychology in a diathesis-stress model, where sensitivity is seen as a vulnerability for developing problematic outcomes (*diathesis)* when faced with adversity (*stress*) (61). This perspective stipulates that some individuals are disproportionately likely to succumb to the negative effects of contextual stressors. However, this model makes no claims about differences in how resilient or vulnerable individuals develop under favorable conditions. A more recent theoretical framework, the differential susceptibility hypothesis (DSH), suggests individuals differ in environmental sensitivity more generally, not just in the response to negative stressors (62).

In contrast to the diathesis-stress model, which implies a definite and mostly negativity-sensitive group, Belsky et al. describe a group that is sensitive to negative experiences but also positive experiences as “for better AND for worse” (62). The DSH suggests that natural selection drives and maintains these two developmental strategies, which are fundamentally different in susceptibility. The plastic strategy, high susceptibility, is characterized by adaptation to the environment, while the fixed strategy, low susceptibility, reflects little or no reactivity in response to the environment. In resilience research, this theory is important to consider because, on some occasions, those carrying a “protective” allele may function worse than others when not exposed to the risk condition being studied. The DSH also allows for the possibility that some genetic variants may moderate the effects of both positive and negative environments on resilience (63, 64). For example, the same genetic variants that may increase the risk of mental health problems when faced with adversity appear to make individuals more likely to benefit from positive environmental factors. This has important implications for genetic studies of resilience because if individuals are less reactive, i.e., experience little or no psychopathology despite high risk, they could be defined as resilient. Yet a specific reactivity phenotype could be adaptive and confer resilience in some conditions. Very few studies to date capture truly comprehensive measures of the environment, longitudinally and across the complete spectrum from adverse to positive, which may explain why there is such inconsistency in candidate GxE findings (42).

Defining resilience as merely the lack of psychopathology would mean resilience is a passive process, yet most resilience research now points to it being an active, dynamic process (65). Resilience is not only the absence of psychopathology but also positive adaptation (25), although this area is less well-studied. In humans, studies range from measuring self-reported resilience, referring to appraisal, and the concept of functioning resiliently in the face of stress. Despite these being different aspects of resilient phenotypes, the main aim is to find underlying genetic variants in order to identify general resilience mechanisms.

## Present Study

To our knowledge, despite the recent advances and reviews on the genetics of resilience (5, 66), no comprehensive, interdisciplinary systematic reviews have been conducted to examine the genetic variants associated with resilience in humans and animals. Due to the complexities of definition, the potential for common resilience mechanisms to share a variety of outcomes, and the importance of animal studies in the search for biological mechanisms of resilience, this review incorporates general inclusion criteria for the definition of resilience in both humans and animal models. It is important to note that we do not seek to develop a novel definition of resilience. Rather, we include all previously published research that have defined resilience in different ways in order to identify general resilience mechanisms and underlying genetic variants.

The main aim of this review is to identify genetic variants associated with resilience. Identifying common genetic variants associated with resilience in humans and animals will provide construct validity to both animal models and human studies of resilience. Identifying genetic variants associated with resilience as a measure of both reactivity and positive adaptation will provide support for the idea of a general resilience mechanism.

## Method

This review has been conducted following PRISMA guidelines and guidance by Sagoo et al. (67) on systematic reviews of genetic association studies (67).

A systematic search of EMBASE, Medline, and PubMed was performed in October 2021 with the terms “resilien^*^” AND “genotype” in search category: title, abstract, and full text. The wildcard operator (^*^) was used to include all possible suffixes (*resilience, resilient, resiliency)*. In addition, research articles associated with a phenotype of “Resilience, Psychological” in the HuGE (Human Genome Epidemiology) Navigator Phenopedia (68) were included. Published years were not restricted.

**Figure 1:**
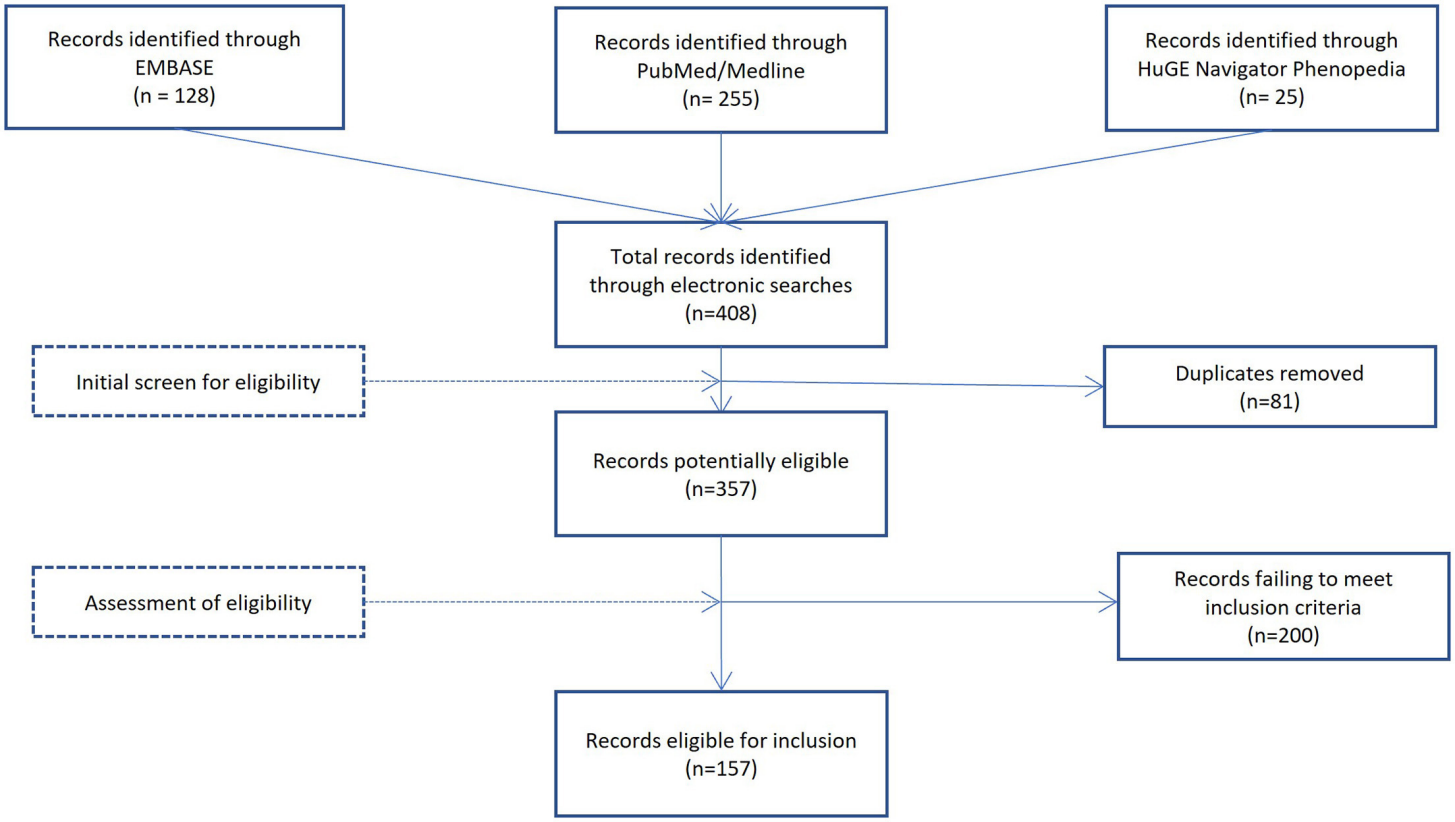
Identification and selection process. The figure shows the sources used for identification and assessment of eligibility of studies for inclusion in this review with number of studies in brackets.

To be included in this review, manuscripts had to be written in English, peer reviewed, and report a significant association between a genetic variant and an outcome associated with resilience (either positive adaptation or reactivity). DNA methylation, twin, and heritability studies of resilience were excluded from this review because they do not identify specific genetic variants associated with resilience. Titles and abstracts were initially screened to determine eligibility. If questions remained about the eligibility, the entire manuscript was reviewed to ascertain inclusion. This resulted in 158 studies, including 30 studies identified through references of the records identified through the electronic search. Author 1 conducted the review, with authors 2 and 3 systematically cross-checking the data-extraction process. See Figure 1 for the selection process.

### Data Extraction

The following information was extracted:

Human studies: (1) Population characteristics (number of patients vs. controls, age range, sex, adult or child population, important characteristics), (2) resilience measurement, (3) candidate gene, (4) resilient allele (if any), (5) environmental risk factor (if any), (6) key findings.

Animal studies: (1) Species, (2) population characteristics (age, sex, transgenic, KO, subpopulations), (3) candidate gene, (4) environmental stressor (if any), (5) key findings.

All data extraction can be found in Supplementary Table 1.

## Results

The systematic searches initially generated 408 titles, that were reduced to 327 after removal of duplicates. An additional 30 studies were identified through reference lists, leaving 357 full-text articles assessed for eligibility. In total, 156 papers were selected for this review. The selected papers were then separated into three areas, (1) animal studies of resilience (*n* = 28), (2) human studies with resilience measured by positive adaptation (*n* = 51) and, (3) human studies with resilience measured by level of reactivity (*n* = 77). A total of 62 candidate genes were associated with resilience. Descriptions of the functional importance for each gene are summarized in Table 1.

**Table 1:** Gene functions for each gene identified in the systematic review with two or more studies reporting association with resilience.

**Gene**	**Function**	**No. of Studies**
*5HTT, SLC6A4*	The *5-HTTLPR* is a length polymorphism (VNTR) in the serotonin transporter (*SLC6A4)* gene promoter region with two main alleles. A single base substitution in the long form of *5-HTTLPR* (rs25531 A/G) and the short allele of *5-HTTLPR* have been associated with decreased serotonin transporter availability and resulting inhibition of serotonin reuptake from synaptic clefts (69). HTTLPR is functionally triallelic: the L allele with a common G substitution, L(G), creates a functional AP2 transcription-factor binding site, and thus has functional variants designated as L(A), L(G), and S. The L(G) and S alleles have comparable levels of serotonin transporter expression (69). There is a common, functional polymorphism, rs25532, located <150 nucleotides centromeric of *5-HTTLPR*, whereby the minor allele of rs25532 significantly decreases luciferase reporter gene expression levels, dependent on *5-HTTLPR* allele background and type (70).	36
*BDNF*	Brain-Derived Neurotrophic Factor (*BDNF*) encodes a member of the nerve growth factor family of proteins. A SNP, rs6265, at nucleotide 196(G/A) produces an amino acid substitution, valine to methionine, at codon 66 (*BDNFVal66Met* genotype). Met-carriers of this polymorphism have decreased secretion of *BDNF* (71).	14
*COMT*	Catechol-O-methyltransferase (*COMT*) is an enzyme that inactivates catecholamines, including neurotransmitters dopamine, epinephrine, and norepinephrine (72). A SNP, rs4680, produces an amino acid substitution, valine (Val) to methionine (Met), at codon 158 (Val158-Met). The Met/Met homozygote is associated with a difference in thermostability, leading to a three-four fold reduction in the activity of the *COMT* enzyme in catabolising dopamine, epinephrine, and norepinephrine. The Val/Val homozygote has the highest activity of *COMT*, the Met/Met homozygote has the lowest activity, and heterozygous individuals are intermediate (73).	9
*DRD4*	The dopamine receptor D4 gene (*DRD4*) carries a variable number tandem repeat (VNTR) polymorphism, which is repeated between 2 and 11 repeats in the third exon (exIII). DRD4 variants carrying seven copies of the tandem repeat have significantly suppressed expression of the reporter compared to those carrying four and two copies, likely *via* mechanisms involving translational efficiency or RNA stability (74). However, this relationship was not supported in later research using post mortem brain tissue (75). In addition, a SNP in *DRD4*, rs1800955, describes −521 C/T, which is a Cytosine (C) to Thymine (T) transition at base −521 in the upstream promoter region. The −521C allele was found to increase the transcriptional efficiency of *DRD4* by 40% in cultured cells (76), however this was not confirmed in later research (77, 78).	5
*OXTR*	The oxytocin receptor gene (*OXTR*) is a protein that belongs to the G-protein coupled receptor family and acts as an oxytocin receptor (72). A SNP, rs53576 (major allele: G, minor allele: A), which maps to the 3rd intron (a non-coding region) of *OXTR*, has emerged as a promising candidate genetic marker of individual differences in social behavior, stress reactivity, and psychopathology (79). In addition, a SNP in the third intron, rs2254298, has been identified as a promising candidate to explain differences in oxytocinergic functioning (80).	6
*APOE*	Apolipoprotein E (*APOE*) encodes a protein that is a major apoprotein of the chylomicron and is essential for the normal catabolism of triglyceride-rich lipoprotein constituents. The human apoE protein has three primary isoforms— ε2, ε3, and ε4— that differ in terms of the presence of cysteine or arginine at two positions (residues 112 and 158) of the 299 amino acids that comprise the protein, resulting in six possible genotypes: ε4/ε4, ε4/ε3, ε4/ε2, ε3/ε3, ε3/ε2, and ε2/ε2. These differences affect the structure and function of the apoE isoforms (72, 81).	6
*NPY*	Neuropeptide Y *(NPY)* encodes a neuropeptide that is widely expressed in the central nervous system, and its pleiotropic functions comprise of cortical excitability, stress response, food intake, metabolism, and vascular and immune function (41, 72, 82, 83). A single promoter variant, rs16147, affects gene expression (83) and accounts for approximately half of the variance in a haplotype analysis, showing that a five marker haplotype spanning the NPY promoter predicts central NPY mRNA levels (41). The C-allele is associated with increased NPY gene expression in the anterior cingulate cortex (82).	7
*TNFα*	*TNFα* encodes a pleiotropic proinflammatory cytokine, mainly secreted by macrophages, that belongs to the tumor necrosis factor (TNF) superfamily. *TNFα* signals through two transmembrane receptors, TNFR1 and TNFR2, and is involved in the regulation of several critical biological processes, including cell proliferation, differentiation, apoptosis, lipid metabolism, and coagulation (72, 84). Several biallelic SNPs have been noted in the *TNFα* gene (84), including rs1799964 and rs2229094. Among them one functional polymorphism (rs1800629) is located upstream of the gene at −308 and is known to influence TNF-α levels. In comparison with the *TNF-α* −308G allele, the A allele has higher transcriptional activity (85).	5
*FKBP5*	FKBP prolyl isomerase 5 *(FKBP5)* is a co-chaperone of the hsp90, which regulates glucocorticoid receptor (GR) sensitivity. Polymorphisms in *FKBP5* (e.g., rs1306780, rs9296158, and rs9470080) are associated with differential expression of *FKBP5* and resulting in variation in GR resistance and stress hormone system regulation (86).	5
*MAOA*	Monoamine oxidase A (*MAOA)* is located on the X chromosome (Xp11.23) and encodes mitochondrial enzymes, which catalyse the oxidative deamination of amines, such as dopamine, norepinephrine, and serotonin (72). A commonly studied functional polymorphism is *MAOA* gene exon 8 rs6323 (T941G) has been related to high (941G) and low (941T) *MAOA* enzyme activity. The G allele results in increased amine degradation and decreased availability of neurotransmitters such as dopamine, norepinephrine and serotonin. The T allele leads to decreased amine degradation (87).	3
*SLC6A3 (DAT1)*	The dopamine transporter (*DAT1)* gene plays a role in dopaminergic neurotransmission by mediating the reuptake of synaptic dopamine back into the neurons. It contains a 40-base-pair VNTR in the 3′ untranslated region (UTR) of the gene (*SLC6A3*). The number of repeat sequences ranges between 3 and 13 copies, with the 9- and 10-repeat alleles the most common (88, 89). Meta-analyses investigating the functional effects of *DAT1* genotypes on *in vivo* dopamine transporter functioning provide significant evidence that the 9R allele is associated with increased DAT activity in humans (90, 91).	3
*CRHR1*	Corticotropin-releasing hormone receptor 1 *(CRHR1)* encodes a G-protein coupled receptor that binds neuropeptides of the corticotropin-releasing hormone family and is widely distributed through the brain (92). A distinct TAT haplotype formed by the three most significant *CRHR1* SNPs (rs7209436, rs110402, and rs242924) has been found to affect stress-system processes including cortisol response (93), although the specific effects of the TAT polymorphisms on *CRHR1* functioning have not been elucidated.	3
*CACNA1C*	Calcium voltage-gated channel subunit alpha1 C *(CACNA1C)* is the gene that encodes the Cav1.2 subunit of L-type voltage-gated calcium channels. This subunit mediates the influx of calcium ions into the cell and initiates downstream signaling cascades (94). A SNP at rs1006737 is considered a functional polymorphism: individuals with the A/A genotype are associated with increased CACNA1C messenger RNA expression in the pre-frontal cortex, compared with A/G or G/G genotypes (95).	2
*IL10*	Interleukin 10 (*IL10)* encodes a pleiotropic cytokine produced primarily by monocytes and some lymphocytes. *IL10* plays a central role in immunoregulation and inflammation (72). *IL-10* exhibits high polymorphisms with as much as 75% of inter-individual variability in human IL-10 expression attributed to genetic variations, with SNPs rs1800896, rs1800871, and rs1800872, at positions −1,082 [G/A], −819 [C/T], and −592 [C/A], respectively, the three most commonly studied (96, 97).	2
*MTHFR*	The methylenetetrahydrofolate reductase (MTHFR) encodes a protein that catalyses the process of one-carbon metabolism involving folate and homocysteine metabolisms (72). A common SNP polymorphism, A1298C/rs1801131 A/C, is found in the coding carboxy-terminal regulatory region domain (98), with the 1,298 (A > C) transition resulting in decreased MTHFR activity *in vitro* (99).	2
*OPRM1*	Opioid receptor mu 1 (*OPRM1)* shows an increased affinity for the endogenous ligand, ß-Endorphin. MORs are involved in positive reinforcement following direct or indirect activation. The SNP rs1799971 is an A-G substitution that results in a functional amino acid substitution, Asn40Asp (100). The 118A > G allele has been associated with opioid and alcohol addiction and variations in pain sensitivity but there is conflicting evidence for its causal role (72).	2
*CRHBP*	Corticotropin-releasing hormone binding protein (*CRHBP*) is a potent stimulator of synthesis and secretion of proopiomelanocortin-derived peptides (72). Corticotropin-releasing hormone (CRH), a primary physiological mediator of the HPA axis, is regulated by *CRHBP* by preventing CRH from binding its receptors. The two CRHBP SNPs, rs1500 and rs7728378, that have been associated with resilience in this review are located at the 3′ end of the gene but no evidence of functionality has been reported.	2
*IL6*	Interleukin 6 (*IL6)* encodes a pleiotropic cytokine that has multiple functions in immunes and hematopoietic activities. *IL6* is primarily produced at sites of acute and chronic inflammation, where it is secreted into the serum and induces a transcriptional inflammatory response through interleukin 6 receptor, alpha (72). IL-6 levels increase over the lifespan and up-regulate in response to stress. A polymorphism in the promoter sequence of the IL6 gene (rs1800795; IL6–174G/C) modulates individual sensitivity to the effects of stress in activating *IL6* transcription (101).	2
*RNASE13*	Ribonuclease A family member 13 (inactive) (*RNASE13)* is a protein coding gene with low expression in the reference dataset (72). Two SNPs, rs3748348 and rs3648346 have been associated with executive functioning resilience in GWAS but have not had biological activity mapped.	2

*We provide the gene name, followed by its function and the number of studies that meet the inclusion criteria and have an established association of the gene with a measure of resilience in humans*.

### Alleles Associated With Resilience as Measured by Positive Adaptation (*N* = 51)

Resilience is measured using a number of different instruments and measurement tools as highlighted in Table 2.

**Table 2:** Operationalisation of resilience as a form of positive adaptation and measurement tools utilized.

**Resilience conceptualization**	**Measurement tool**	**References**
Resilience scale (RS)	Connor-Davidson resilience scale (CD-RISC) − 25 or 10 item questionnaire measuring resilience *via* a range of coping strategies shown to be successful mediators in dealing with adversity	(37, 102–113)
	RS-25—a 25 item questionnaire with higher scores indicating greater perceived resilience	(114–116)
	Shortened Greek RS-14	(117)
	Shortened RS-15	(118)
	Chinese adapted brief resilience scale—six items describing one's ability to bounce back from stress	(119)
	Study to assess risk and resilience in service members (STARRS) 5-item self-report questionnaire on ability to handle stress in various ways	(17)
	27 item resilience scale of the traumatic brain injury quality of life (TBI-QOL)	(120)
Sense of Coherence scale (SOC) − 29-item semantic differential questionnaire referring to a consistent but dynamic feeling of confidence, defined as the belief that life is comprehensive, manageable, and meaningful	Original version 29 item	(121)
	3-item Swedish SOC version	(122)
	SOC-L9—unidimensional short version	(123)
Dispositional optimism	Life orientation test	(110, 124)
	Optimism subscale of the Dutch scale of subjective wellbeing for older persons	(125)
	Positive future focus measured by the future orientation scale	(119)
Positive affect	Subscale of the positive affect and negative affect scale for children	(59)
Distress intolerance	Behavioral indicator of resilience to distress (BIRD)	(126)
Ego-Resiliency	Adaptability to environmental stress and change using 11-item questionnaire	(115)
Post-traumatic growth (PTG)	21 item PTG inventory. Self-reported positive psychological changes following a traumatic event	(127)
Physical resilience	Physical resilience scale, identifies physical challenges associated with aging	(128)
Stress coping	Japanese ways of coping questionnaire	(129)
	Ways of coping inventory	(130)
Effortful control	11-item subscale of the Dutch version of the revised early adolescent temperament questionnaire	(131)
Attentional control	Ability to inhibit negative information under stress	(132)
	Attentional bias away from negative word stimuli	(133)
	Attentional bias to selectively process positive affective material while avoiding negative affective material	(134)
Secure attachment	Strange situation paradigm used to measure attachment styles in 12-month-old infants	(135)
Neuronal resilience	Post-mortem or temporal lobectomy brain tissues analyzed to assess neuronal resilience to Alzheimer's Disease (AD) and epilepsy	(136–139)
AD resilience	Despite having advanced age and *APOE* ε4 allele, no symptoms of cognitive decline	(140)
Executive function (EF)	Maintenance of high EF performance on neuropsychological tests despite neurodegenerative conditions	(141, 142)
Compute own resilience score	Lifetime trauma exposure (T), and severity of PTSD symptoms (PCL) with ratio of PCL/T indicating sensitivity to trauma and inverse is resilience	(143)
	Composite of resilient functioning from peer, adult, and self-report measures of children functioning well in domains of developmental importance	(144)
	Measured resiliency from victimization by creating a victimization scale, with adolescents asked to indicate the frequency with which they were the victims of five different acts of violence, and creating a composite score of resiliency to victimization longitudinally	(145)
	Utilized growth mixture modeling to identify latent classes of individuals, including those resilient to depression following cancer	(146)
	Composite resilience metrics by summarizing residuals from linear regression models estimating amyloid pathology associations	(147)
	Construct indicators that promote fitness and reflect positive mental and physical health, presence of a supportive social network, and avoidance of incarceration	(148)

Below we discuss, in order of highest frequency, the genes and their function that were identified in two or more studies as being associated with resilience to assess whether consensus is reached on which alleles are associated with resilience.

#### Serotonin Transporter (5-HTT, *SLC6A4*)

The most frequently investigated polymorphism (*n* = 18) was the *5-HTTLPR* (serotonin transporter linked polymorphic region) which is located in the promoter of the human serotonin transporter gene (*SLC6A4*). Out of these studies, 12 found the L′/L′ allele to be associated with resilience (37, 59, 102, 114, 117, 126, 130–134, 144) and four studies found the S or S′ allele to be associated with resilience (103, 104, 123, 145), and one study found that only haplotypes of the serotonin transporter were associated with resilience, the single genetic variants were not (115). There are a several factors that might contribute to these inconsistent findings.

First, the studies used different allelic variation classification. The A 44 bp deletion/insertion results in a short (S) or a long (L) allele, creating a biallelic classification of S/L alleles (149). The rs25531 is a SNP in the L allele, which leads to an A-G polymorphism. This provides a functional triallelic polymorphism as S, L_G_, and L_A_. The L_G_ allele is functionally equivalent to the S allele, having the same expression levels, both of which are lower than L_A_ (69). Consequently, the L′/L′ can be reclassified as equal to L_A_/L_A_; L′/S′ = L_A_/S and L_A_/L_G_; and S′/S′ = S/S & L_G_/S and L_G_/L_G_. This is the triallelic classification system. 12 of the studies excluded the SNP rs25531 from their analyses (37, 102, 103, 114, 115, 117, 126, 130, 132, 134, 144, 145), and five studies genotyped for rs25531 and recoded accordingly (59, 104, 123, 131, 133). One study found a significant association with the biallelic classification system, but not with the triallelic (37), and another study excluded the results of the triallelic system due to a low number of participants carrying the L_G_ allele (102).

A second factor contributing to inconsistencies in findings between studies are differences in the developmental stage at which effects were studied. Eight of the studies investigated children or adolescents, while ten investigated adults. Within the studies that did use the triallelic classification system, those that found that the L/L or L′/L′ genotype of *5-HTTLPR* contributed to resilience, investigated children/adolescents (59, 131, 133), while those that found the S/S or S′/S′ genotype investigated adults (104, 123). This could be explained by the evolutionary genetic theory of antagonistic pleiotropy (150), which was first proposed as an explanation for multiple effects of a gene with opposing effects on fitness depending on the developmental stage. Notably, genes enhancing reproductive success in early life are predicted to become deleterious in later life. This opposite effect in young vs. old participants should be interpreted with caution because, although there is some evidence emerging of the age-genotype interaction [e.g., (151, 152)], the evidence is limited.

A third factor that may explain discrepancies in consensus are sex differences in the *5-HTTLPR* polymorphism. Previous research from both animals and humans suggests that males and females carrying the short *5-HTTLPR* allele respond to environmental stress factors in opposite directions (153, 154). A recent review of sex differences in affective disorders found the S allele to be differentially associated with an increased risk of depression, anxiety, and internalizing behavior in women, and increased risk of aggressiveness, conduct disorder, and externalizing behavior in men (155). The two studies that investigated female-only populations (114, 132) found the L allele to be associated with resilience, while one study investigating males only found the S/S homozygous genotype to be associated with resilience.

#### Brain-Derived Neurotrophic Factor

The second most frequently investigated gene was Brain-Derived Neurotrophic Factor (*BDNF)* (*n* = 5). All five of the studies reviewed investigated SNP rs6265 in exon 11 of the *BDNF* gene (105, 116, 122, 129, 131). Of the four studies, three found Val/Val homozygous genotype to be associated with resilience, while two studies found the Met allele to be associated with resilience. This inconsistency in results could, again, be due to a number of confounds. Allele frequencies of the *BDNF* Val66Met polymorphism are dependent on ethnic background: frequency of the Met allele among Caucasians is 25–32% but is more common in Asians at 40–50% (156). Of the five studies, Aizawa et al. (129) and Kang et al. (105) both studied Asian populations, Nederhof et al. (131) and Surtees et al. (122) both studied European Caucasians, and Peters et al. (116) studied Brazilian nationals. Sex differences in the populations studied may also result in inconsistent findings. A recent review found some action mechanisms or functions of *BDNF* vary according to sex and encourage considering sex influences when drawing conclusions from studies (157). One study reviewed only observed a significant association between resilience and the Val allele in males (105).

Two of the studies assessed polygenic resilience with a gene-gene interaction effect. One study suggests a sex-dependent result, where the *BDNF* Val66Met polymorphism modulates the effects of the Val158Met polymorphism of *COMT* on resilience, but only in males (105). The other study investigated the gene-gene interaction between *BDNF* Val66Met polymorphism and *5-HTTLPR*, specifically investigating resilience depending on the genotype of the other gene variant (131).

#### Oxytocin Receptor Gene

Three studies investigated the rs53576 polymorphism in the oxytocin receptor gene (*OXTR)*. Cicchetti and Rogosch (144) found the impact of child maltreatment on resilient functioning was decreased in children with the GG genotype when compared to GA or AA genotypes, assuming the dominant effect of the A allele. Saphire-Bernstein et al. (124) reported a link between the GG genotype and higher levels of optimism, mastery, and self-esteem, suggesting that the influence of this *OXTR* polymorphism on psychological resources may primarily mediate the effects of *OXTR* on depressive symptoms.

In contrast, Bradley et al. (158) reported that, regardless of family environment, adults with the AA genotype had higher levels of positive affect and resilient coping scores. The authors reported that GG and AG genotypes were more influenced positively and negatively by the family environment, in keeping with the differential susceptibility hypothesis.

One study investigated the *OXTR* variant rs2254298 on psychological resilience as measured by CD-RISC (106). The authors found a dose-dependent effect of the A allele in ethnically Korean adolescents and young adults, with highest resilience in carriers of the GG genotype, decreasing by 3.84 on the CD-RISC scale with a one-copy increase in the A allele. An *OXTR* haplotype containing the rs2254298 A allele was also associated with lower CD-RISC scores.

#### Apolipoprotein E

Four studies investigated Apolipoprotein E (*APOE*). The presence of the ε4 allele is a strong genetic risk factor for Alzheimer's disease (AD), whereas the ε2 allele is protective against AD. Two of the studies found both the ε3 and ε2 allele to be associated with resilience (136, 143), and the other study found ε3 allele to be associated with resilience (137). However, this study did not genotype for the *APOE* ε2 allele, so there is consensus amongst the studies reviewed about which allele is associated with resilience despite the studies using different instruments to measure resilience and associating the allele with different outcomes: AD, PTSD, and epilepsy. A fourth study found US veterans with mild traumatic brain injuries (TBIs) had significantly better scores on the resilience scale of the TBI- Quality of Life measure (120), but again, the study did not genotype for ε2 or ε3 isoforms of *APOE* so it cannot be included in the consensus of the *APOE* ε2 allele conferring resilience.

#### Catechol-O-Methyltransferase

Three studies investigated catechol-O-methyltransferase (*COMT)*. All studies investigated a functional polymorphism on rs3680, Val^158^Met (105, 107, 126), yet again the results were inconsistent. Of the two studies which used the same instrument to measure resilience, the CD-RISC, van Rooij et al. (107) found childhood trauma load was associated with increased inhibition-related hippocampal activation in Val/Val carriers, with the relationship between childhood trauma and resilience mediated by hippocampal activation. This implies that Val/Val carriers may develop a mechanism to cope with early adversity by relying more on contextual information processed in the hippocampal region in order to regulate behavior. However, Kang et al. (105) found a conflicting, polygenic, and sex-dependent result. Males with homozygous Val allele of *BDNF* and *COMT* Met-present genotype were found to have higher resilience than *BDNF* Met carriers. Amstadter et al. (126) also found associated resilience with the Met allele. This inconsistency could again be attributed to sex differences: van Rooij et al. (107) only studied women, while Kang et al. (105) only found a significant association with resilience in males. *COMT* enzyme activity and the neurochemistry and behavior of *COMT* knock out mice are both markedly sexually dimorphic (159). In addition, the Val^158^Met polymorphism shows sexual dimorphism in predisposition to psychiatric disorders (159).

Results could also be conflicting due to the different ethnic groups analyzed by the researchers. *COMT* Val/Met allele frequencies differ across ethnic groups, with higher heterozygosity in European populations than other geographical regions (160).

Further the assumed mode of inheritance in each study may contribute to inconsistencies in results. Both Kang et al. (105) and van Rooij et al. (107) assumed dominance of the Met allele and grouped the sample into subjects with the Val/Val genotype vs. Val/Met and Met/Met. By contrast, Amstadter et al. (126) assumed dominance of the Val allele (Met/Met vs. Val/Met and Val/Val). Due to this difference in modes of inheritance, it is unclear whether heterozygotes of rs4680 in *COMT* (Val/Met carriers) would be associated with increased or decreased resilience.

#### Dopamine Receptor D4

The human dopamine receptor D4 gene (*DRD4*) was investigated by three studies (108, 135, 144). Das et al. (108) found an interaction effect of childhood adversity, the *DRD4* 7-repeat allele, and resilience. A decline in resilience was associated with increased childhood adversity in individuals who lacked the 7-repeat allele, therefore suggesting that the 7-repeat allele is protective. Conflicting results come from Gervai's et al. (135) study, which analyzed a haplotype of the 7-repeat allele and the −521C/T promoter polymorphism of *DRD4* which found an absence of the T.7 haplotype acts as a resilience factor. Cicchetti and Rogosch (144) studied the effects of the −521C/T promoter polymorphism of *DRD4* (rs1800955) and revealed another interaction effect where maltreated children with CC or CT genotypes were protected from the significantly detrimental effect of extensive maltreatment, assuming the dominant effect of the C allele (CC and CT vs. TT). This is in some way consistent with Gervai et al. (135).

#### Tumor Necrosis Factor α

Two studies investigated tumor necrosis factor α (*TNF*α) (113, 146). Bruenig et al. (113) examined a SNP (rs1800629) in the promoter region of *TNF*α, which has been identified with potential functional consequences for gene transcription. While no significant effect was observed between CD-RISC and genotype, there was a significant correlation between resilience and decreased *TNF*α serum levels. Dunn et al. (146) found two SNPs to be significantly associated with a resilient latent class: rs2229094 and rs1800629. Individuals who possess the A rare allele for rs1800629 had higher odds of belonging to the resilient class, while individuals who possess the T allele in rs2229094 had higher odds of belonging to the resilient class.

#### Interleukin-10

Interleukin-10 (*IL10*), was associated with resilience in two studies (110, 146). Rana et al. (110) found the SNP rs1800896 in *IL10* to be associated with optimism in an exploratory multi-locus polygenic analysis with two other SNPs: rs6323 of *MAOA* and rs1800792 of Fibrinogen Gamma Chain (*FGG)*. Dunn et al. (146) found participants who possessed a G allele of SNP rs1518111 of *IL10* to have higher odds of belonging to the resilient latent class. Both studies used a strategy of selecting high-probability candidate genes to analyse. While scientifically justifiable, selecting genes from a list of “usual suspects” introduces bias, and many genetic variants identified by GWAS have included genes previously not thought to be involved in the target disease etiology (161).

#### Ribonuclease a Family Member 13

Two studies, although of the same population, found two significant genome-wide associations between SNPs (rs3748348 and rsrs3648346) of Ribonuclease A Family Member 13 (*RNASE13)*, and executive functioning resilience (141, 142), suggesting a cognitive basis for resilience. Pathway-based analysis by gene set enrichment analysis implicated EF resilience with genetic pathways involving dendritic/neuron spine, presynaptic membrane, and post-synaptic density (142). *RNASE13* has been linked to susceptibility for aortic aneurysm in Japanese individuals (162).

#### Monoamine Oxidase A

Two studies investigated monoamine oxidase A (*MAOA*) genetic variants (110, 163). In addition, Azadmarzabadi et al. (111) examined mRNA levels of *MAOA* and found that *MAOA* expression is downregulated in individuals with low stress resilience, as measured by the CD-RISC. Rana et al. (110) found SNP rs6323 in *MAOA* to be associated with both resilience and optimism in an exploratory multi-locus polygenic analysis. Tikkanen et al. (163) found alcoholic violent offenders carrying the low MAOA activity genotype (3-repeat allele) had no increase in risk of reoffending when exposed to the independent predictors of recidivism, heavy drinking and childhood physical abuse. Significant associations have previously been reported between *MAOA* and anger-related traits (164).

### Alleles Associated With Resilience as Operationalised by Levels of Reactivity (*N* = 77)

The 77 studies that found alleles associated with resilience *via* an array of measures that associated with some level of reactivity to the environment are shown in Table 3. Despite these wide ranges of operationalising resilience, they will be examined together with the goal of finding shared biological pathways.

**Table 3:** Operationalisation of resilience as measure of reactivity, the outcome measured, and measurement tool utilized.

**Level of reactivity**	**Outcome measured**	**Measurement tool**	**References**
No or mild psychopathological symptoms despite increased risk	Internalizing problems	Lower internalizing symptoms *via* childhood behavior check list (CBCL)	(165)
		Child and adolescent symptom inventory	(166)
		Youth self report	(167)
	Externalizing problems	Child and adolescent symptom inventory	(166)
	Social support	Networks of relationships inventory	(167)
	Anxiety	Decreased risk of generalized anxiety disorder (GAD)	(168, 169)
		Anxiety sensitivity index	(170)
		Anxiety diagnosis by DSM-IV in suicide attempters	(171)
		Stait-trait anxiety inventory	(82)
		Hospital anxiety and depression scale (HADS)	(172, 173)
		Combat experiences log	(174)
		Spielberger state anxiety inventory	(175)
		Spielberger trait anxiety inventory	(175)
	Depression	Resilient to depression risk in terms of cortisol and recent stress	(176)
		Childhood depression inventory (CDI)	(58, 177, 178)
		DSM-IV major depression disorder (MDD) diagnosis	(179)
		Geriatric depression scale	(180, 181)
		Beck depression index	(82, 175, 182–185)
		Sadness subscale of affective neuroscience personality scales (ANPS)	(186)
		Center for epidemiological studies depression scale	(174, 187)
		Montgomery–Åsberg depression rating scale	(175)
	Pathological worry	Penn state worry questionnaire	(170)
	Performance impairment	Psychomotor vigilance impairment following total sleep deprivation	(188–190)
	Behavioral problems	Conduct problems: theft, truancy, suspension, fighting	(191)
		Emotional and social problems as measured by strength and difficulties questionnaire	(192)
		Conscientiousness *via* big five factor scales	(193)
		The adaptive and maladaptive impulsivity scale	(175)
		Neuroticism	(175, 194)
		Severe impulsive acts of violence among alcoholic violent offenders	(163)
	Post-traumatic stress disorder (PTSD)	Fear extinction deficits	(195)
		PTSD checklist from DSM-V and DSM-IV	(174, 196–200)
		Diagnostic interview schedule-III-revised (DIS-III-R)	(201)
	Schizophrenia risk	DSM-IV schizophrenia diagnosis	(202)
	Psychosis risk	Prodromal questionnaire brief	(203)
	Stress	Subjective stress—Groningen acute stress test	(204)
		Stress reactivity conceptualized as psychotic reactivity to daily life events and minor disturbances in daily life, recorded *via* ESM (experience sampling method)	(205)
		Psychological stress	(206)
		Social stress sensitivity on residual depressive symptoms	(207)
		Recent stressful life events prior to admission for depressive episode	(208)
	Suicide	Suicide attempt	(209)
	Emotion regulation	Executive function: heart & flowers task that assesses inhibition, working memory, and cognitive flexibility	(210)
		Emotion regulation questionnaire for children and adolescents	(58)
		Emotion dysregulation scale	(211)
	Attachment	Indiscriminate social behavior *via* disturbances of attachment interview	(212)
		Child attachment security	(58)
		The influence of early maternal care on fearful attachment	(213)
Neural correlates	Brain activation	Amygdala and ventromedial prefrontal cortex activation/responsiveness during fear acquisition and extinction	(214)
		Brain activation elicited by affective stimuli and cognitive tasks	(215)
		Regional responses to emotional faces in the amygdala and subgenual cingulate cortex	(216)
		Amygdala and prefrontal cortex stimulation to standardized affective visual stimuli	(217)
		Ventral striatal activity to the angry faces task	(192)
		Impact of serum klotho levels on measures of greater intrinsic connectivity in key functional networks of the brain vulnerable to aging and AD such as the fronto-parietal and default mode networks	(218)
	Regional volumes	Differences in dorsolateral prefrontal gray matter (GM) volume	(219)
		GM volume alterations conditional on adversity	(220)
		GM volume alterations associated with schizophrenia	(40)
		Hippocampal and whole brain volumes in Alzheimer's disease (AD) patients	(221)
		Hippocampal atrophy in in cognitively normal patients with APOE ε4 risk allele.	(222)
	White matter abnormalities	Fractional anisotropy in the right parietal lobe of schizophrenic patients and controls	(223)
Decreased likelihood of illness/mortality despite high risk	Alzheimer's disease	Reduced incidence of late onset AD despite carrying high risk APOE ε4 allele	(224)
		Clinical dementia rating sum of boxes and digit span forwards scores in AD patients	(221)
		Cognitive decline in cognitively normal patients with APOE ε4 risk allele	(222)
	Leukemia	Reduced risk of developing adult leukemia	(225)
	Lifespan	Longevity despite cardiovascular disease	(226)
		Longevity in individuals with hypertension	(227)
		Longevity despite cardiometabolic disease	(228)
Proxy measures of reactivity	Cortisol response	Saliva cortisol was measured during and after the trier social stress test for children	(229, 230)
		The role of early life stress on the cortisol awakening response	(231)
	Heart rate variability	Differences in vagal tone under chronic stress conditions	(232)
	C-Reactive protein	Heightened inflammation-related disease risk associated with adverse socio-environmental conditions	(101, 151)
	Cerebrospinal fluid (CSF) volumes	CSF t-tau and p-tau levels	(221)
	Serum Klotho levels	Relationship between genotype and systemic klotho levels	(218)
Abstinence, decreased dependency or risk of addiction, despite high risk	Problem alcohol use	Alcohol use disorders identification test	(233)
		Risk of alcoholism identified using the substance abuse section of the structured clinical interview for DSM-IV	(234)
	Heroin addiction	Met DSM-IV criteria for substance dependence	(235)
		Prolonged abstinence without methadone treatment	(236, 237)
	Opioid dependence	Daily injectors vs. opioid misusers who didn't progress to injection	(238)

#### Serotonin Transporter (5-HTT, *SLC6A4*)

The most frequently investigated polymorphism associated with the operationalisation of resilience as levels of reactivity, was again the *5-HTTLPR* (*n* = 18). The environmental measures included stressful life events, stroke, war zone combat, victimization, racial discrimination, child abandonment, interferon-alpha treatment, and neuroticism. Of these 16 studies, 13 found the L allele to be associated with resilience (132, 148, 165, 166, 174, 180, 182, 191, 194, 196, 212, 214, 216), whereas three studies found the S allele to be associated with resilience (177, 197, 203), and two studies found the SL heterozygous allele to be associated with resilience (176, 220).

#### Apolipoprotein E

Two studies investigated *APOE*, both concerning the severity of PTSD symptoms among veterans exposed to combat trauma (198, 201). Both studies found that absence of the ε4 allele was associated with significantly less severity of PTSD symptoms than carriers of the ε4 allele.

#### Brain-Derived Neurotrophic Factor

Eight studies investigated the *BDNF* Val66Met polymorphism (172, 173, 178, 179, 200, 203, 212, 219) in environmental measures of childhood adversity, childhood abandonment, traumatic life events, newly diagnosed diabetes, and physical exercise. Of the eight studies, 4 associated the Met allele with resilience (178, 179, 203, 212), and the other three found Val/Val or Val/Met genotype to be associated with resilience (172, 173, 200, 219). Interestingly, the four studies that found the Met allele to be associated with resilience all studied children or young adults. Min et al. (172) found a gender-specific effect with the Val/Met genotype in males appearing to be resilient against the higher levels of anxiety associated with exposure to childhood maltreatment. Mata et al. (178), also found gender-specific effects where being physically active was protective for girls with a *BDNF* met allele against the development of depressive symptoms, but not for girls with a Val/Val genotype—examining how physical activity contributes to resilience to psychopathology.

#### Catechol-O-Methyltransferase

Five studies investigated *COMT* polymorphisms, and all studies reported the Val allele as associated with resilience, reaching consensus (186, 204, 215, 217, 229). Of the five studies, two studies were fMRI studies assessing the effects of *COMT* Val158Met genotype on emotional stimuli processing. They found the presence of a Val158 allele results in an increase in emotional resilience against negative mood states *via* decreased neuronal activation to unpleasant stimuli (215, 217). Two studies used psychological measures; subjective stress experience (204), and sadness score (186), and one study assessed resilience *via* cortisol response levels (229).

#### Corticotropin-Releasing Hormone Receptor 1

Five studies investigated the interaction between child abuse/maltreatment and polymorphisms in the corticotropin-releasing hormone receptor 1 gene (*CHRH1*). *CRHR1* is a major regulator of neuroendocrine, autonomic, and behavioral responses to stress, and its ligand, *CRH*, has been associated with mood and anxiety disorders (239). The studies found significant GxE interactions with multiple individual SNPs, as well as with a common three allele haplotype, the TAT haplotype, formed by SNPs rs7209436, rs110402, and rs242924. Bradley et al. (240) found that carriers of the TAT haplotype were found to be protected from the adverse impact of severe childhood abuse, as measured by the Childhood Trauma Questionnaire (CTQ). In an extended sample of the Bradley et al. study, Heim et al. (241) confirmed the protective effect of the rs110402 A-allele against the negative emotional consequences of childhood abuse in the male subsample only. They hypothesize that this effect may not be due to sex differences but due to the differences in the abuse experienced, with physical abuse most commonly experienced by men, and sexual abuse most commonly experienced by women. Although, Polanczyk et al. (242) only replicated the protective effect of the TAT haplotype in a sample of women. The second sample in Polanczyk's et al. study consisted of men and women, and no significant result was found. The authors suggest that this difference is attributable to the different types of measurement of maltreatment between the samples, with maltreatment reported *via* the CTQ more likely to elicit emotional memories, and the hypothesized link between *CRHR1*'s protective effect is related to its function in the consolidation of memories of emotionally arousing experiences.

Grabe et al. (183) identified a previously unreported SNP, rs17689882, in which the minor allele (A) had a protective effect against depressive symptoms among individuals who had experienced moderate to severe neglect. Laucht et al. (184) replicated this result, finding the minor A allele as protective.

#### Dopamine Transporter 1

Two studies investigated GxE interactions associated with the dopamine transporter 1 gene (*DAT1, SLC6A3*). Felten et al. (186) found carriers of the homozygous 9-repeat allele showed dramatically reduced sadness scores in a sample of healthy Caucasians. In contrast, Satterfield et al. (188) found that subjects homozygous for the 10-repeat allele were resilient to the build-up of cognitive performance impairment due to total sleep deprivation.

#### *FKBP* Prolyl Isomerase 5

Three studies investigated GxE interactions associated with the glucocorticoid receptor co-chaperone, *FKBP5*, which modulates glucocorticoid receptor sensitivity. Two of the three studies examined the moderating effects of *FKBP5* rs3800373 on suicide risk (209) and attachment security and depressive symptoms (58). Roy et al. (209) found that individuals that lacked either major homozygote were resilient to the effects of childhood trauma (suicide attempt prevalence 0.24). Similarly, Borelli et al. (58) found that minor C allele homozygotes had the most optimal psychological profiles in positive caregiving contexts, but the most maladaptive outcomes in adverse caregiving contexts. This is supportive of the DSH. Buchmann et al. (230) investigated the interaction between the *FKBP5* rs1360780 genotype, childhood adversity, and cortisol response. The authors found a blunted cortisol response to environmental challenges, as observed in homozygous carriers of the 9-repeat allele, indicative of an enhanced biological resilience to healthy individuals with a history of childhood adversity.

#### Interleukin-6

Two studies investigated how the *IL6* rs1800795 polymorphism interacts with adverse environmental factors to promote chronic inflammation. The first study found that older adults with a C allele of the polymorphism are protected from the increased risk of inflammation-related disease and mortality shown in individuals with the G allele of the polymorphism and high socioeconomic adversity exposure (101). The second study identified a protective effect of the G allele in adolescents, which desensitizes individual physiology to the pro-inflammatory effects of adverse socioenvironmental conditions (151). This age-dependent antagonistic pleiotropy suggests that genetic resilience is dependent on the developmental environment that interacts with social conditions.

#### Neuropeptide Y

Six studies investigated GxE interactions associated with the polymorphism rs16147 of Neuropeptide Y. Five of the six studies associated individuals homozygous for the T allele with resilience (82, 119, 168, 232). Sommer et al. (82) found that childhood adversity was significantly associated with increased depression and anxiety for homozygote carriers of the C allele, but homozygote carriers of the T allele were protected. Amstadter et al. (168) found the TT genotype interacts with impact level of hurricane exposure to be protective against anxiety. Chang et al. (232) reported an interaction between chronic high stress, the TT genotype, and high cardiac vagal tone, a physiological measure that is gaining support as a marker of resilience by capturing the integration of the varying regulatory mechanisms of the autonomic nervous system (243). Gan et al. (119) reported two separate studies within the same paper. Study 1 found that T allele carrier earthquake survivors reported consistent levels of resilience in low, moderate and high trauma exposure. Study 2 found T allele carriers who reported a high number of early stressful life events were associated with a greater positive future focus.

Donner et al. (169), in contrast to the other studies, found no GxE interaction effect with rs16147, childhood adversity, and anxiety susceptibility, but instead found risk haplotypes significantly associated with anxiety that carry the T-allele of the SNP.

Resnick et al. (118) found an association between NPY and psychological resilience following hip fracture but they did not report allelic variation.

#### Opioid Receptor μ 1

Two studies investigated the A118G (SNP rs1799971) polymorphism of the μ-opioid receptor gene (*OPRM1*). Levran et al. (236) assessed whether opioid-related genetic variants contribute to reduced vulnerability to relapse to heroin in persons without agonist treatment. The authors found that carrying at least one copy of the G allele is significantly higher in subjects with long term abstinence from heroin, suggesting this genotype may blunt the endogenous stress response and contribute to resilience against relapse.

Troisi et al. (213) found that G allele carriers had similarly high scores on fearful attachment, regardless of the quality of maternal care. By comparison, AA genotype carriers were more affected by early experience with those recalling high levels of maternal care exhibiting less fearful attachment behavior, and those recalling low levels of maternal care exhibiting high levels of fearful attachment. This is again in line with the DSH and suggests G allele carriers have a decreased sensitivity to adverse rearing environments.

#### Oxytocin Receptor

Three studies investigated the interactions between polymorphisms in *OXTR* and environments on a range of outcomes. Two of the studies investigated the rs53576 polymorphism in *OXTR*, and both studies associated A allele carriers with resilience. Bradley's et al. (211) findings suggest that A allele carriers are more resilient to the adverse effects of severe childhood adversity by being protected against emotional dysregulation and disorganized attachment. Hostinar et al. (167) replicated this finding, showing maltreated adolescent A-carriers exhibit the same level of psychological symptoms as non-maltreated adolescents, supporting resilience research that the experience of maltreatment does not deterministically lead to psychopathology.

Loth et al. (192) investigated the rs237915 SNP of *OXTR* and found that CC homozygotes were more resilient against the effect of stressful life experiences than other genotypes, partly mediated by genotype-dependent sensitivity to the reinforcement values of negative social cues.

#### Tumor Necrosis Factor α

Two studies investigated the *TNF*α G308A polymorphism on performance impairment during total sleep deprivation (TSD) (189, 190). Skeiky et al. corroborated Satterfield and colleagues' earlier findings that indicated that the A allele at the *TNF*α 308 locus (SNP rs1800629) is associated with resilience to psychomotor vigilance performance impairment during TSD, in comparison with the more common G allele.

### Animal Models of Resilience (*N* = 28)

28 Studies of Animal Models of Resilience met the Inclusion Criteria (Supplementary File 1). Of the 28 Studies, 20 Studies Used Transgenic Mouse Populations, five Studies Used Subpopulations, and three Used Selective Breeding. The Findings of Animal Genes With Relevant Human Homologs Are Discussed Below.

#### Opioid Receptor, Mu 1

Briand et al. (244) used a transgenic mouse model, with C57BL/6 mice possessing the functionally equivalent SNP in the mouse *Oprm1* gene (A112G) as the human *OPRM1* A118G SNP. To determine the role of this polymorphism in behavioral interactions and to investigate the functional mechanisms underlying this behavior, the authors used a social defeat paradigm. The authors found that G-allele carriers were resilient to the deleterious consequences of stress, as measured by positive social interactions and reduced anhedonia. This is consistent with the human studies of *OPRM1* also investigated in this review, which found G-allele carriers are resilient against relapse to heroin use (236) and show decreased levels of fearful attachment in low maternal care environments (213). An increase in brain activation in stress and reward circuitry is also evident in resilient *Oprm1* G/G mice in Briand's et al. (244) study, thus suggesting that modeling this SNP in mice provides construct validity for this model of resilience These models are in direct contrast with results in a recent meta-analysis which found no moderating effect of *OPRM1* A118G, on response to naltrexone treatment in individuals with alcohol use disorder (245).

#### Neuropeptide Y

Cohen et al. examined the *Npy*-ergic system and its association with behavioral responses to stress in a rat model of PTSD (246). The authors classified subpopulations of Sprague-Dawley rats based on their performance in elevated plus maze and acoustic startle response tests using a pre-set cut-off criterion after exposure to predator-scent stress. The study found protective qualities of *Npy*. Notably, an extreme reduction in *Npy* expression in selected brain regions of the animals severely affected by the stressor. This behavioral disruption was reversed by administering exogenous *Npy* directly into the hippocampus of the stressed animals 1 h post-exposure. While the study did not examine genetic variation associated with NPY polymorphisms, the results are still worth noting due to homologous findings in humans.

#### Calcium Channel, Voltage-Dependent, L Type, Alpha 1C Subunit

*CACNA1C* has been identified as one of the strongest genetic risk factors for the development of affective disorders in both GWAS and candidate gene studies, [e.g., (18, 19, 247)]. Three of the 18 animal models examined this gene in mouse models using different approaches.

Terrillion et al. (248) used a subpopulation approach, classifying male wild-type or conditional *Cacna1c* knockout mice on a C57BL/6 background into resilient or susceptible based on their performance in a chronic social defeat test. They found expression of *Cacna1c* in the nucleus accumbens (NAc) is unchanged in resilient mice compared with control mice not exposed to social stress. Susceptible mice show a decrease in *Cacna1c* expression in the NAc after the social defeat paradigm, suggesting normal *Cacna1c* function in the NAc is crucial for resiliency to social stressors.

Dedic et al. (249) used a transgenic mouse model to assess the impact of *Cacna1c* deletion at different stages of development (See Supplementary Table 1 for genetic background and breeding timelines). If embryonic *Cacna1c* deletion in forebrain glutamatergic neurons occurs, then endophenotypes associated with psychiatric disorders manifest themselves. If the deletion of *Cacna1c* from forebrain glutamatergic neurons occurs in adulthood, the opposite effect occurs, inducing improved cognitive flexibility, strengthened synaptic plasticity, and increased stress resilience.

Michels et al. (250) examined a gene-stress interaction using immortalized mouse hippocampal cells, a well-established model system that reflects a common cellular response to environmental stress. In contrast with Terrillion's et al. (248) work, the authors show that reduced *Cacna1c* expression mediates neuroprotective effects against oxidative stress, predominantly at the level of mitochondria. This implies that reduced *CACNA1C* expression converges to control mitochondrial function, resulting in cellular resilience against oxidative stress. There is a growing body of evidence that major psychiatric illnesses are associated with impaired cellular resilience and synaptic dysfunction at various levels (251). These results suggest that alterations in mitochondrial function could affect critical cellular processes, dampening synaptic plasticity, and contributing to the pathophysiology of psychiatric disorders.

Decreased or increased *CACNA1C* expression levels have been associated with the SNP rs1006737, the same SNP investigated in studies included in this review. This, and Dedic's work, suggests that alterations in *CACNA1C* expression may be developmental-stage, brain-region stage, cell-type or sex-specific.

#### Deleted in Colorectal Carcinoma

Manitt et al. used a transgenic mouse model to examine the netrin-1 guidance cue receptor gene, deleted in colorectal cancer (*dcc*), and resilience (252). The *dcc* haploinsufficient male mice were maintained on a BL/6 background and bred with wild-type BL/6 female mice. Using *dcc* loss-of-function transgenic mice, the study found reduced *dcc* expression confers resilience against developing neuroanatomical, neurochemical, and behavioral traits associated with mental disorders involving medial prefrontal cortex (mPFC) dysfunction. Significantly, these protective phenotypes are only present in adult but not pre-pubertal mice and most likely result from selective alterations in the reorganization of mPFC dopamine circuitry. One human study found in this review examined how *DCC* contributes to the risk of schizophrenia (202). The authors found that individuals heterozygous at one SNP, rs2270954, had decreased risk of schizophrenia. Further studies have demonstrated that microRNA regulation of *DCC* by miR-218, a posttranscriptional repressor of *DCC*, maybe a switch of susceptibility vs. resilience to stress-related disorders (253). Together, these studies suggest that *DCC* could be a promising candidate gene that contributes to the genetic basis of resilience.

#### FK506-Binding Protein

Three studies investigated transgenic mouse models of *Fkbp5*, a co-chaperone protein of the Hsp90 complex that regulates the glucocorticoid receptor. *FKBP5* SNPs have been associated with psychiatric disorders including depression, PTSD, bipolar disorder, and anxiety (254). FKBP51, encoded by the *FKBP5* gene, and FKBP52, encoded by the *FKBP4* gene, are components of the chaperone-receptor heterocomplex and differentially regulate the glucocorticoid receptors or mineralocorticoid receptors. Hartmann et al. (255) characterized heterozygous *Fkbp52* knockout mice kept on a mixed 129SvJ × C57BL/6 background, under basal and chronic social defeat stress conditions (CSDS). *Fkbp52* heterozygous knockout mice demonstrated stress resilience, such as reduced basal corticosterone levels and more active stress-coping behavior in the forced swim test (FST) following CSDS. This mimics the phenotype of *Fkbp51* deficient mice in the FST, as found by O'Leary et al. (254). O'Leary et al. tested how *Fkbp5* deletion in aged mice affects behavior, given that FKBP51 levels increase with age. They found that aged *Fkbp51* deficient mice displayed enhanced active stress-coping behavior following the acute severe stress exposure of the FST, similar to levels of control mice treated with antidepressants. This antidepressant behavior in the *Fkbp*5^−/−^ did not affect cognition or other basic motor functions, and reduced corticosterone levels following stress were also observed.

Kwon et al. (256) exposed wild-type and *Fkbp5* knock-out mice to chronic restraint stress. They found *Fkbp5-*deficient mice were resilient to the depressive-like behavior exhibited by wild-type mice following chronic restraint stress. Further RNA sequencing analyses revealed a module of co-expressed genes, M55, that was downregulated in the WT group and restored in the KO group. Gene ontology enrichment analysis of M55 revealed biological functions involved with inflammatory response, gland morphogenesis and nervous system development.

Together, these findings suggest that absence of FKBP5 and regulation of the immune response results in a decrease of corticosterone levels and HPA-axis activity, which increases resilience to depressive phenotypes.

#### Leucine-Rich Repeat Kinase 2

Depression has been reported to be more common in patients with Parkinson's disease (PD) than in the general population, often appearing earlier than the onset of motor symptoms (257). The G2019S mutation in *LRRK2* causes late-onset PD, but it is unclear how this mutation impacts depression-related behaviors (258). Matikainen-Ankney et al. (258) addressed this by subjecting *Lrrk2*-G2019S knock-in and wild-type mice to CSDS. Mice used in the study were congenic on C57BL/6NTac background. The study showed that young adult G2019S knock-in mice were highly resilient to CSDS, failing to exhibit social avoidance compared to wild-type mice. There were no behavioral differences between the genotypes found in the absence of CSDS.

#### Apolipoprotein E

Given the well-documented research on the *APOE* ε4 genotype association with genetic risk for Alzheimer's Disease (AD), and human imaging studies suggesting *APOE* ε4 is associated with brain structures linked to cognitive decline, Kulkarni et al. (259) assessed brain structure and function in *APOE* ε4 knock-in rats. Male and female WT and human *APOE* ε4 knock in Sprague Dawley rats were studied for changes in brain structure and behavioral tests. There was evidence of sex differences in brain structure and function in the *APOE* ε4 knock in rats with the data suggesting female carriers are more resilient to cognitive/emotional problems possibly due to altered brain volumes and enhanced connectivity.

## Discussion

Our review of 157 articles revealed 62 genes that were empirically associated with resilience in humans and animal models (Supplementary Table 1). Despite the complexities of defining the concept of resilience, enormous heterogeneity in the way resilience is defined, operationalised and measured, and the way empirical resilience research is designed and conducted, we found that several genetic variants are consistently associated with resilience (Table 4).

**Table 4:** Genetic variants associated with resilience in humans and animals reaching consensus.

**Gene**	**Allele**	**Study designs and resilience concepts**	**References**
*SLC6A4*	L/L or L′/L′	Studies investigating children, using the triallelic classification system with resilience operationalised as positive affect, effortful control, and attentional control	(59, 131, 133)
*SLC6A4*	S/S or S'/S'	Studies investigating adults, using the triallelic classification system with resilience operationalised by the CD-RISC and RS-25 resilience scales	(104, 123)
*APOE*	ε3	Studies measuring resilience as decreased sensitivity to trauma, and neuronal resilience to AD and epilepsy	(136, 137, 143)
*BDNF*	Met	Studies investigating children measuring resilience as low levels of indiscriminate social behavior, lack of MDD diagnosis, or depressive symptoms	(178, 179, 212)
*COMT*	Val	Studies measuring resilience using cortisol response following stress as a proxy for resilience; low sadness scores; decreased limbic activity to unpleasant stimuli; and reduced level of subjective stress	(186, 204, 215, 217, 229)
*CRHR1*	A	Both studies using different populations found the impact of child adversity on adult depression scores reduced in the minor allele (A) carriers of rs17689882	(183, 184)
*OPRM1*	G	At least one copy of 118G is significantly higher in subjects in long term abstinence from heroin and G allele carriers exhibited less fearful attachment behavior in the face of aversive maternal care environments	(213, 236)
*OXTR*	A	A allele carriers of rs53576 are resilient against the effects of severe childhood adversity, by protection against emotional dysregulation and disorganized attachment and maltreated A carriers perceived higher social support, had lower levels of internalizing symptoms and were indistinguishable from non-maltreated adolescents in level of mental health symptoms	(167, 211)
*TNFα*	A	Individuals who possess the A rare allele of rs1800629 has significantly higher odds of having a resilient depressive symptom trajectory following cancer and was associated with greater resilience to psychomotor vigilance performance impairment during total sleep deprivation	(146, 189, 190)

*After the gene name we identify which allele is associated with resilience as defined in the respective studies, followed by a summary of the study designs that found the same alleles associated with resilience, the number of studies that identified an association with resilience, and the references*.

Notably, *OPRM1, NPY, CACNA1C, DCC, APOE*, and *FKBP5* had both animal and human variants linked to measures of resilience. This supports the idea of shared biological pathways of resilience. The A118G polymorphism in *OPRM1* stands out because in all studies carriers of the G-allele were classified as resilient despite completely different environmental measures and outcomes. This suggests that the A118G polymorphism in *OPRM1* could be a novel therapeutic target for further investigation. A recent meta-analysis and systematic review indicates that G allele carriers of *OPRM1* A118G required more opioid analgesia in pain management (260), and *COMT* haplotypes are also associated with pain sensitivity and opioid efficacy (261). Pain perception and opioid response are complex traits and might involve interactions between these or multiple genes (262), important for future gene network research. Evidence suggests that variation in *OPRM1*, as measured by the A118G polymorphism, is associated with individual differences in rejection sensitivity (263), suggesting an overlap between physical and social pain. A systematic review found pain perception to be a resilience factor directly salient to physical illness (16).

*DCC* also stands out as a promising candidate gene that contributes to the genetic basis of resilience. In addition to the results of the candidate gene studies included in this review, in both human and animal studies that identified associations between variation in *DCC* and resilience, *DCC* also shows well-established associations with schizophrenia (264), MDD (265) and cross-disorder risk (21), intelligence (266), cognitive ability and educational attainment (267), noting that the latter two measures have wide support as individual resilience factors. These genome-wide significant findings set *DCC* apart from those that are mainly supported by small candidate gene studies (regarded as problematic in the field). Thus, the association between *DCC* and resilience warrants further investigation.

The rs16147 SNP in human *NPY* reported in this review influences hypothalamic–pituitary–adrenal (HPA)-axis responsiveness to acute psychosocial stress (268). Further studies in humans have demonstrated that genetic variations in the *NPY* locus, especially in the promoter region, substantially mediate *NPY* release, with low *NPY* expression predicting lower resilience (41, 269). This suggests that *NPY* is a particularly plausible candidate for modulating effects of environmental stress exposure on resilience as supported by the rat model in this review and other animal models (270, 271). Higher *NPY* plasma levels have also been found in veterans resilient to PTSD (272), potentially representing a biological correlate of resilience to, or recovery from, the adverse effects of stress. Recent systematic reviews and meta-analyses have found *NPY* variation to interact with childhood trauma to influence anxiety sensitivity (273) and the risk of obesity (274). Resilience, specifically emotional and family resilience, are protective factors against obesity in children (275, 276), suggesting interaction effects between *NPY*, resilience and obesity.

What inferences can we make about resilience by these genetic association results? Although not all studies reach consensus on alleles associated with resilience, sex differences are an important consideration for several reasons. First, *COMT* enzyme activity and the neurochemistry and behavior of *Comt* knock out mice are both markedly sexually dimorphic (159). Sex effects were also found in *BDNF* and mouse models of *APOE*. Clinical evidence supported by rodent and human studies suggests that females are more vulnerable to drug addiction than males (277). It is further important to consider sex effects within environmental exposure measures. For example, males will be more likely to be exposed to physical abuse, and females will be more likely to be exposed to sexual abuse. Some of the studies investigated in this systematic review had only male participants or female participants [e.g., (107, 132)]. For the results of these studies to be generalized, they need to include both sexes, or be investigated separately.

Another important consideration in these candidate gene studies is sample size. The sample size of the human studies ranged from *n* = 12 to *n* = 7,335 so the vast majority were severely underpowered (278, 279) suggesting high false discovery rates for the positive associations reported here. Despite this, the consensus of the genetic variants repeatedly associated with resilience, in its many forms, warrants further investigation. We would particularly recommend whole genome data analyses of resilience, where resilience is quantified using a residuals approach, as recommended by emerging frameworks [e.g., (6, 280)].

### Genetic Associations of Resiliency Phenotypes

Due to the vast array of resiliency phenotypes, as shown in Tables 2, 3, we analyzed the current literature to assess if the resiliency phenotypes in our systematic review were found to be associated with overlapping candidate genes in recent meta-analyses, GWAS or systematic reviews. We used the resiliency phenotypes where resilience was operationalised as positive adaptation.

#### Longevity

Longevity was used as a resilience phenotype in three studies (226–228) and has also been previously associated with individuals with protective variants, buffering the effect of disease-associated variants and adverse environmental factors, decreasing morbidity at the end of extreme longevity (29–32). A recent review and meta-analysis confirmed the association of five polymorphisms that were significantly associated with exceptional longevity: *ACE* rs4340, *APOE* ε2/3/4, *FOXO3A* rs2802292, the *KLOTHO* KL-VS variant and *IL6* rs1800795 (281). Of these five polymorphisms, three had genetic associations with resilience within our review, with resilience conceptualized as decreased sensitivity to trauma (143), neuronal resilience to AD (137), epilepsy (136), elevated serum klotho levels that promote a resilient brain (218), and desensitized physiology to pro-inflammatory effects (101, 151).

#### Sense of Coherence

Sense of coherence, referring to a consistent but dynamic feeling of confidence, defined as the belief that life is comprehensive, manageable and meaningful, was operationalised as a resilience phenotype in three studies (121–123). The first GWA analysis recently conducted for SOC yielded a significant association, rs74920024 on chromosome 2 (282). The association locus is close to Glycerol-3-Phosphate Dehydrogenase 2 (*GPD2)*, a gene encoding a protein that localizes to the inner mitochondrial membrane and is responsible for the conversion of glycerol-3-phosphate to dihydroxyacetone phosphate. While there is no overlap of this gene with candidate genes found in this review, *GPD2* has been previously associated with large GWAS's on cognitive ability (283) and educational attainment from the Social Science Genetic Consortium 2018 GWAS (267), and cognitive aspects of educational attainment from a large GWAS meta-analysis combining a cognitive consortium and human intelligence GWAS (284). While these traits have not been used as a proxy for resilience within this review, they have wide support as individual level resilience factors [e.g., (285–287)], with high cognitive skills in childhood predicting resilience to adversity in adolescence (288).

#### Coping

Two studies within this review operationalised resilience as stress coping (129, 130). Çarkaxhiu Bulut et al. (130), evaluated the *SLC64A* 5-*HTTLPR* polymorphism and resilience components on the development of psychopathology in adolescent sexual abuse cases, while Aizawa et al. (129) examined genetic association of *BDNF* and variation in stress-coping style. A recent systematic review of genetic influences on coping (289) found multiple associations between *SLC64A* and Adrenoceptor Beta 2 *(ADRB2)* and coping phenotypes. One important finding of their review that echoes the sentiments within our own review, is the lack of consensus regarding instruments for coping phenotypes. Again, despite the lack of consensus of instruments within the reviews, there is overlap on the candidate genes associated with coping and resiliency phenotypes. A study included in this review found a novel association of a SNP within the promoter region of the *ADRB2* gene that was associated with relative resilience to childhood adversity (199). Previous research has revealed links between *ADRB2* and resting blood pressure (BP) and regulation of BP in stressful situations (290, 291). This genetic association of *ADRB2* found in both coping and resiliency phenotypes is interesting given the known links between stress and risk factors for cardiovascular disease.

#### Attachment

Gervai et al. (135) examined the link between *DRD4* gene polymorphism and infant attachment and found that not carrying the T.7 haplotype of the *DRD4* acts as a resilience factor in the optimal development of early attachment. Within a recent systematic review of gene-environment interactions on infant and child attachment (292), *DRD4* was associated with the interaction between maternal sensitivity and parenting style in five studies, with contradictory results. While there was also a lack of consensus of which *DRD4* allele was associated with resilience in the studies meeting our selection criteria in this review, later research found the *DRD4* 7-repeat allele to be protective against the risk of a dysfunction in emotional regulation associated with birthweight (293). This is consistent with Das's et al. (108) finding but contradictory to Gervai's et al. (135) and Cicchetti and Rogosch's (144). While these associations are suggestive of reoccurring patterns of associations, ultimately, there is insufficient evidence to meaningfully comment on the direct influence of *DRD4* on attachment or resilience, a position reiterated in a later systematic review of the genetics of adult attachment (294).

#### Post-traumatic Growth

While the conceptualization of resilience in the literature is highly variable, the relationship between PTG and resilience is not clear. Some researchers equate resilience with PTG (295), while others assume it is a form of resilience (296). Only one study included in this review met the inclusion criteria (127) and examined the association between PTG, PTSD and genetic variants. The study found a GxE association between PTG and Regulator of G Protein Signaling 2 (*RGS2*) (rs4606), driven by disaster exposure. However, a recent systematic review found no gene expression changes associated with PTG (297), again highlighting a focus on pathogenic research, which fails to adequately measure or acknowledge positive post-trauma outcomes, whether they be conceptualized at resilience or PTG.

### Assessing the Validity of Animal Models of Resilience

Future work focusing on shared biological pathways between the candidate genes highlighted in this review may reveal common biological mechanisms of resilience. Revealing these mechanisms would bring us closer to understanding the process of adapting to aversive stimuli and could reveal new therapeutic targets for symptom reduction. Animal models of disease therefore provide an ethically and economically viable experimental platform to examine these common biological mechanisms of resilience and identify biomarkers that may facilitate the development of resilience to adversity, or reduce the symptoms of post-adversity outcomes. It is therefore crucial that future animal models of resilience are judged by specific validity criteria. While currently no validity criteria for animal models of resilience exist, animal models of psychiatric disorders use validity criteria following Willner (298). He defined three specific multidimensional criteria that must be met for an animal model of a psychiatric disorder to be considered relevant for human pathology: face validity, predictive validity and construct validity (298). The first criterion, face validity, is considered as the degree of phenomenological similarity between the model and the phenotype being modeled. This phenomenological identity encompasses behavioral and cognitive aspects only, not just their physiological or neural bases. For resilience, face validity would be whether the animal can avoid some of the deleterious behavioral effects of chronic stress and is thus less prone to exhibit maladaptive behavioral traits. This would mimic diagnostic criteria such as those in DSM-5, where criteria are generally behavioral or cognitive, and a threshold for diagnosis is when such symptoms cause significant impairment in the individual's ability to function.

The second criterion, predictive validity, corresponds to human-animal correlations of therapeutic outcomes. While the criterion of predictive validity initially relied on a pharmacological correlation (i.e., the animal model shows the same response to a specific treatment as humans), a more recent paper by the same author (299), extended the criterion to include the response to all available treatments, not just pharmacological treatments. For example, within the depression model, this means not just pharmacological antidepressants, but also electroconvulsive therapy. Extending to models of resilience, there are behavioral treatment options for enhancing resilience in humans and animals using non-pharmacological treatments, such as stress inoculation (300), exposure therapy/fear extinction (301) and cognitive behavioral therapy (302). There is limited research on predictive validity of pharmacological correlations of animal models of resilience. However, using the chronic social defeat stress paradigm, Wilkinson et al. (303) found a significant overlap in genes that are regulated in resilience phenotypes and those that are regulated by chronic antidepressant treatment of susceptible individuals, with Holanda et al. (304) repeating this finding with acute and prolonged swimming stress. This suggests that resilient individuals may be less vulnerable to the adverse effects of chronic stress by exhibiting the same changes in specific expression profiles in response to stress as those induced by antidepressants. Experimentally, evaluating which genetic variants determine an animal's capacity for resilience could allow for predictive validity of animal models *via* new treatment options for stress-related disorders.

Construct validity, the third criterion, refers to the assumption that the behavior of the animal model is homologous to the behavior seen in humans. Ideally, construct validity in animal models of resilience would be achieved by replicating in an animal the etiologic processes that create a resilient phenotype in humans. One of the biggest challenges for establishing construct validity within animal models is the absence of reliable genetic markers of resilience (305). Since *OPRM1, NPY, CACNA1C, DCC*, and *FKBP5* had both animal and human variants linked to measures of resilience this provides some construct validity for these animal models of resilience. The *OPRM1* mouse model of resilience is the strongest candidate to provide construct validity of resilience because in all studies, carriers of the G-allele were considered resilient. By incorporating animal models in the study of resilience in this review, we maximized construct validity of animal models of resilience by focusing on homologous resilience alleles that underlie general resilience mechanisms in both humans and animals.

### Genetic Background in Model Systems

Similarly to how consideration of ethnicity is important in human studies (156, 160), the genetic background (e.g., mouse or rat strain) of animal models of resilience is critical in determining the response to stressors. Increasing evidence suggests that genotype-phenotype relationships cannot be reliably inferred by studying a singular genetic background, with genetic background dramatically altering experimental conclusions (306, 307). An example of this is the *Cacna1c*^+/−^ phenotype. Our review found three animal studies reporting a positive association with *Cacna1c* and an outcome associated with resilience (248–250). Yet research on F1 crosses between male C57BL/6J mice heterozygous for null alleles of *Cacna1c* and wild-type females from 30 inbred laboratory strains, found the majority of null phenotypes were not generalisable (307). Phenotypic responses strongly interacted with the genetic background of the mouse strain, with some responses drawing diametrically opposed conclusions on the same allele. The choice of genetic background in animal models of resilience is critically important because genetic mutations in some strains can lead to the disruption of genes crucial in certain biological processes (308). In our review, Briand et al. (244) found G-allele carriers of C57BL/6 mice possessing the functionally equivalent SNP in the mouse *Oprm1* gene (A112G) as the human *OPRM1* A118G SNP, resilient to the deleterious consequences of stress. Future animal studies modeling this SNP in this mouse model should carefully select the background: DBA/2J possess coding and non-coding variants in *Oprm1* and therefore exhibit weaker morphine preference and response compared with C57BL/6J (309, 310). Known mutations in genes that are relevant to the phenotype-of-interest, in this case human G-allele carriers of *OPRM1* resilient to heroin relapse (237), must be avoided in order to reduce unwanted bias. These results do not negate the invaluable contributions of mouse genetics to biomedical science, but they highlight that in order to reveal the functional biological networks underpinning resilience, we must broaden our focus beyond single genotype models and instead leverage the opportunities available using advanced genetic models such as recombinant inbred strains (311, 312).

### Developmental and Epigenetic Aspects

The developmental time frame during which gene expression is modulated needs to be considered due to the vital influence of developmental timings on maturation of different parts of the stress system on later stress vulnerability or resilience (50). As seen in Table 4, both *SLC6A4* and *BDNF* have alleles associated with resilience when the populations are either adults or children. This can be explained by age-dependent antagonistic pleiotropy, suggesting that genetic resilience is dependent on the interaction between developmental stage and social conditions. This is again supported by an animal model in this review showing the opposite impact of *Cacna1c* deletion on psychopathology and cognitive flexibility at different stages of development (249), with a recent review also showing gene expression in tissues is time dependent (313).

The consideration of developmental timeframe effects is of paramount importance in the DSH framework. One view of the DSH could be that genetic variants are not predefined as resilience or even risk factors, but often they determine both risk and resilience dependent on the current context of gene-environment interaction. This conceptualization could also offer significant insight into the many discrepancies in this review regarding which allele is associated with resilience. According to the DSH, specific polymorphisms would be characterized by differential environmental sensitivity, enhancing positive outcomes in positive environments, and elevating vulnerability in adverse environments. Indeed, five studies investigated within this review report findings consistent with the DSH (58, 144, 168, 212, 213).

When investigating underlying biological mechanisms of resilience, epigenetic mechanisms such as DNA and histone modifications, and non-coding RNAs are critical in regulating cellular function and systemic physiology through their impact on gene expression (312). Such mechanisms are emerging as key mediators of environmental factors (313–315), providing a potential pathway through which individuals modify their individual responsiveness to a stressor, which may in turn contribute to individual resilience (316). Many epigenomic modifications are dynamic and reversible and may also provide targets for intervention strategies. For example, a recent systematic review of DNA methylation studies in PTSD, PTG and resilience found that high resilience was associated with a blunted immune response (297). While our aim for this review was to identify genetic variants associated with resilience, the incorporation of epigenetic variants and markers will be an important consideration for future research into resilience factors.

## Conclusion

Despite limitations linked to the complexities of defining resilience, the vast array of measurement instruments, outcomes, and populations investigated, 62 candidate genes that met the inclusion criteria have been identified, and 22 studies reach consensus on which allele confers resilience. Based on these findings, future work should focus on shared biological pathways between these candidate genes, which may reveal common biological mechanisms of resilience. This would support the omnigenic model (317): genetic variants underlie biological processes, not diagnoses, and the same processes play a role in different manifestations of resilience. We recommend longitudinal studies of genetic variants of *OPRM1, OXTR, CRHR1, COMT, BDNF, APOE*, and *SLC6A4* in human and animal models with consideration of developmental timing, type and severity of stressor, sex and ethnicity.

## Data Availability Statement

The original contributions presented in the study are included in the article/Supplementary Material, further inquiries can be directed to the corresponding author.

## Author Contributions

SC defined the research question, performed the systematic review and analyzed the articles. TC and RH systematically crosschecked the review. SC wrote the manuscript. Substantive edits and revisions were conducted by RH and SC. All authors read and approved the final manuscript.

## Funding

SC was supported by ESRC grant ES/P000347/1 Soc-B (Social-Biological) Center for Doctoral Training: UCL-Manchester-Essex Consortium.

## Conflict of Interest

The authors declare that the research was conducted in the absence of any commercial or financial relationships that could be construed as a potential conflict of interest.

## Publisher's Note

All claims expressed in this article are solely those of the authors and do not necessarily represent those of their affiliated organizations, or those of the publisher, the editors and the reviewers. Any product that may be evaluated in this article, or claim that may be made by its manufacturer, is not guaranteed or endorsed by the publisher.
